# Normalization of impaired emotion inhibition in bipolar disorder mediated by cholinergic neurotransmission in the cingulate cortex

**DOI:** 10.1038/s41386-022-01268-7

**Published:** 2022-01-19

**Authors:** Leila Nabulsi, Jennifer Farrell, Genevieve McPhilemy, Liam Kilmartin, Maria R. Dauvermann, Theophilus N. Akudjedu, Pablo Najt, Srinath Ambati, Fiona M. Martyn, James McLoughlin, Michael Gill, James Meaney, Derek Morris, Thomas Frodl, Colm McDonald, Brian Hallahan, Dara M. Cannon

**Affiliations:** 1 Center for Neuroimaging, Cognition and Genomics (NICOG), Clinical Neuroimaging Lab, NCBES Galway Neuroscience Centre, College of Medicine, Nursing, and Health Sciences, National University of Ireland Galway, H91 TK33 Galway, Ireland; 2 Imaging Genetics Center, Mark and Mary Stevens Neuroimaging & Informatics Institute, University of Southern California, Marina del Rey, CA 90292 USA; 3 College of Engineering and Informatics, National University of Ireland Galway, Galway, Ireland; 4 Department of Forensic and Neurodevelopmental Sciences, Institute of Psychiatry, Psychology and Neuroscience, King’s College London, London, SE5 8AF UK; 5 Institute of Medical Imaging & Visualisation, Bournemouth University, Bournemouth Gateway Building, St Paul’s Lane, Dorset, BH12 5BB UK; 6 Department of Psychiatry, School of Medicine, Trinity College Dublin, Dublin, Ireland; 7 Department of Psychiatry and Psychotherapy, Otto-von-Guericke-Universität Magdeburg, University Hospital Magdeburg, Magdeburg, Germany

**Keywords:** Bipolar disorder, Diagnostic markers

## Abstract

The muscarinic-cholinergic system is involved in the pathophysiology of bipolar disorder (BD), and contributes to attention and the top-down and bottom-up cognitive and affective mechanisms of emotional processing, functionally altered in BD. Emotion processing can be assessed by the ability to inhibit a response when the content of the image is emotional. Impaired regulatory capacity of cholinergic neurotransmission conferred by reduced M_2_-autoreceptor availability is hypothesized to play a role in elevated salience of negative emotional distractors in euthymic BD relative to individuals with no history of mood instability. Thirty-three euthymic BD type-I (DSM-V-TR) and 50 psychiatrically-healthy controls underwent functional magnetic resonance imaging (fMRI) and an emotion-inhibition paradigm before and after intravenous cholinergic challenge using the acetylcholinesterase inhibitor, physostigmine (1 mg), or placebo. Mood, accuracy, and reaction time on either recognizing or inhibiting a response associated with an image involving emotion and regional functional activation were examined for effects of cholinergic challenge physostigmine relative to placebo, prioritizing any interaction with the diagnostic group. Analyses revealed that (1) at baseline, impaired behavioral performance was associated with lower activation in the anterior cingulate cortex in BD relative to controls during emotion processing; (2) physostigmine (vs. placebo) affected behavioral performance during the inhibition of negative emotions, without altering mood, and increased activation in the posterior cingulate cortex in BD (vs. controls); (3) In BD, lower accuracy observed during emotion inhibition of negative emotions was remediated by physostigmine and was associated with cingulate cortex overactivation. Our findings implicate abnormal regulation of cholinergic neurotransmission in the cingulate cortices in BD, which may mediate exaggerated emotional salience processing, a core feature of BD.

## Introduction

Bipolar disorder (BD) is a severe and burdensome psychiatric condition involving recurrent depressive and manic episodes and overall instability in regulating emotions [1]. The severity and burden of bipolar affective dysregulation affects the quality of life of approximately 1–3% of the world population [2]; however, the neurobiological basis leading to the illness remains unclear. Current pharmacological intervention of BD includes mood stabilizers and antipsychotics; however, the time lag in efficacy for these drugs, or inefficiency [3], makes BD treatment suboptimal resulting in a proportion of patients remaining refractory to treatment. Hence, there is a greater need for the discovery of new agents that are specific to biologically defined streams within BD. Using pharmacological neuroimaging research, it is important to identify neural biomarkers with predictive validity for therapeutic response to enhance diagnostic specificity and guide treatment decisions, to identify the different biological subtypes of BD that may exist and to address the biological heterogeneity extant within mood disorders.

Emotion processing involves the detection and evaluation of salient stimuli as well as the regulation of one’s emotional (affective) response to these stimuli [4]. Dysregulated emotional responses can lead to pathological mood states. Among the array of emotion-related impairments in BD, a hallmark of this disorder is the distorted information processing or attentional allocation toward emotional stimuli [5]. In particular, the salience of negative stimuli is proven challenging in life for those presenting with the disorder [6]. Impaired ability to inhibit a response to emotional stimuli (i.e., the emotion-inhibition condition of the functional task in this study) is one aspect of emotion regulation that may be caused by distorted perception and exaggerated responses to emotional stimuli [7]. Emotion processing can be assessed by the ability to inhibit a response when the content of the image is emotional - the emotion-inhibition condition of the fMRI task assessed herein. Although these are clinically and functionally relevant key features of manic and depressive states in BD, and to a lesser extent of euthymia [7], the neural mechanisms that underpin these impairments are poorly understood. Relating clinically meaningful endpoints to neurotransmitter system circuits is feasible using pharmacological challenge during functional task-based MRI and has the potential to inform more precise symptom management.

Much evidence converges in implicating a molecular signature in human emotion processing, with specific neuromodulatory systems modulating task-specific cognitive states and emotions [8]. In terms of BD, human emotion processing is relevant in the following context. The hypothesis that the brain’s cholinergic neurotransmitter system could act as a modulator of depression and mania was originally proposed in the 1970s by Janowsky and colleagues in their cholinergic-adrenergic hypothesis of mania and depression [9], recently revisited [10], whereby increased and decreased central cholinergic states in the brain could contribute to low and elevated mood states, respectively. Behaviorally the cholinergic system plays major roles in attention, and the top-down and bottom-up mechanisms of emotion processing [11–13]; cognitive mechanisms known to be functionally impaired among individuals with in BD [4]. Numerous lines of evidence have since shed further light on the role of the cholinergic system in mood-regulation and specifically for the muscarinic-cholinergic system in BD, drawing upon post-mortem, genetic, pre-clinical and clinical studies [14].

Studies using adequate doses of cholinomimetic agents such as physostigmine have been shown to exacerbate depressive signs and symptoms in actively depressed subjects [15], and in euthymic individuals with BD [15–17]; as well as psychiatrically-healthy individuals [15–18]. These effects are mediated centrally as administration of simplified analogues of physostigmine not crossing the blood-brain barrier, such as neostigmine, do not result in these depressive effects [19]. Crucially, these effects are mediated by muscarinic receptors [20, 21] as they are reversed by antimuscarinic agents such as biperiden [22]. Furthermore, cholinomimetic drugs, including physostigmine have been demonstrated to play a role in the reversal of mania in BD [23–27]. It is noteworthy that various monoamine transmitters may interplay with the muscarinic-cholinergic system in mechanisms of depression and mania, implying a muscarinic-cholinergic modulatory effect on mood [28]. Further evidence for cholinergic system involvement in BD comes from in vivo molecular positron emission tomography where Cannon and colleagues [29] showed that the muscarinic-cholinergic type-2 receptor (M_2_), an inhibitory or autoreceptor, is less available in the limbic system and particularly in the cingulate cortices in BD relative to major depressive disorder or controls [29]. In a follow-up study, Cannon and colleagues [30] suggested that changes in M_2_-receptor availability may be led by a variation in the gene (CHRM2) coding for the M_2_-receptor (TT genotype of SNP rs324650). Cannon and colleagues [30] in their study of BD using [18 F]FP-TZTP radioligand, sensitive to intersynaptic concentrations of ACh, suggest that the observations of reduced PET binding in BD subjects may be attributable to differences either in M2-receptor density or affinity, or intrasynaptic ACh concentrations. In recent large-scale GWAS studies, several polymorphisms of the CHRM2 gene have been associated with features of cognitive decline in healthy controls [31], though not rs324650 specifically. Although large-scale GWAS studies have not yet identified cholinergic genes as prominent risk factor for BD, evidence for cholinergic genes involvement in BD illness have been derived from small scale studies [6].

BD is associated with structural and functional abnormalities that overlap with regions involved in ventromedial and ventrolateral routes to emotional control [1, 32, 33]. Studies focusing on functional MRI (fMRI) activation in euthymic BD in relation to emotion processing reported increased activation in frontolimbic regions (including amygdala, nucleus accumbens and prefrontal cortex [34, 35]) and decreased activation in the basal ganglia (caudate, putamen and globus pallidum [36, 37]) and in the inferior and middle frontal cortices [36–39]. These areas overlap with the ventral-dorsal systems thought to underpin the processing of emotion and participate in circuits responsible for attributing and perceiving the salience of emotional information [40, 41]. These areas are rich in cholinergic projections [42] and overlap anatomically with regions of previously implicated low muscarinic-cholinergic receptor distribution volume in BD [29]. Taken together with the robust pharmacological evidence for mood effect of cholinomimetics agents reviewed earlier it is clear that the muscarinic-cholinergic system is implicated in aspects of BD illness.

In a randomized, double-blind placebo-controlled study using the acetylcholinesterase inhibitor physostigmine during an emotion-inhibition fMRI paradigm, we aimed to demonstrate muscarinic-cholinergic involvement in clinically relevant behavioural features of BD relative to a control group. We examined behavioural performance (accuracy and reaction time) and related neural abnormalities during processes of emotion recognition and emotion inhibition. Our hypothesis is that subjects with BD will display reduced capacity for inhibitory regulation of muscarinic-cholinergic neurotransmission upon stimulation of the system with physostigmine relative to placebo and control groups. Further, that physostigmine would impact behavioural performance (accuracy and reaction time) in terms of inhibition of negative emotional stimuli, and that the effects observed in BD will exceed the expected effects in healthy controls. We did not anticipate that physostigmine would significantly alter mood at the given dosage of 1 mg, considering worsening of mood had been observed only at higher dosages in both euthymic and actively depressed BD subjects [15, 16]. The expected impact of this research is to inform on the contribution of the muscarinic-cholinergic neurotransmitter system to BD; and highlight the relevance of developing therapeutics targeting this system to ameliorate core emotional clinically relevant features of BD.

## Materials and methods

### Emotion recognition and inhibition task

In BD, there is evidence that cognitive performance is impaired when there are emotional stimuli in the environment. Participants were trained on the task prior to MRI scanning; participants were first asked to stare at a fixation cross, then asked to process visual stimuli with different emotional valence, followed by a question referring either to the emotional valence of the picture (the emotion-recognition trial) or to the image orientation (the emotion-inhibition trial). The emotion-recognition trial involved participants to focus on the emotional content of the picture, namely if the image had positive, negative or no (neutral) emotional valence. The image orientation trial involved participants to focus on the physical orientation of the frame of the picture, namely if the image was in portrait (vertical) orientation or not. Participants answered ‘yes’ or ‘no’ using their dominant hand on a two-button response box (Current Design Inc, USA). Participants did not know which of the trial types they would be asked, which forced them to process both types of information (emotion and orientation), ultimately having to inhibit one type depending on the question asked. Accuracy (number of accurate hits) and reaction times (ms) were collected (Presentation Software, Neurobehavioral Systems 18.0 v, Neurobehavioral Systems, Inc., Berkeley, CA, www.neurobs.com). To remove outliers, in each fMRI task accuracy and reaction time were thresholded at the individual level. For reaction times (RT), a selective mean was calculated as the overall mean_RT_/standard deviation_RT_ and only those values falling within ± two standard deviations from the selective mean were extracted for each event type; in other words, subjects who were too fast or too slow at answering the question asked were considered outliers and thus not analyzed. Based on this RT threshold, accuracy was extracted for each event type, i.e. the number of hits for those subjects considered outliers (based on RT) were not analyzed.

### Study design & pharmacological challenge

This study was designed as a randomized double-blind placebo-controlled trial involving subjects undergoing fMRI task-based imaging (acquisition and task detailed below) before and after a pharmacological challenge of the cholinergic system. After the first fMRI task session and 30 min prior to the start of the second task session, participants underwent intravenous infusion of placebo (sodium chloride 0.9%) or physostigmine, at an initial dose of 2 mg/hr for 10 min followed by 0.8 mg/hr to completion of the study resulting in a maximum possible dose of 1 mg physostigmine [43]. Glycopyrrolate 0.2 mg *i.v*. was administered before physostigmine infusion to minimize peripheral side effects of physostigmine [44]. At this dose and rate of infusion physostigmine has been demonstrated to have minimal side effects while altering behavioural performance to a detectable level [11].

### Study participants diagnosis & genotype

Participants aged between 18 and 65 years were recruited from the western regions of Ireland’s Health Services via referral (outpatients) or public advertisement (patients and controls). All participants were genotyped (detailed below) for the muscarinic-cholinergic type-2 receptor gene (CHRM2) single nucleotide polymorphism (SNP) rs324650 and matched for genotype (AA, AT and TT) within diagnostic groups. Groups were matched for age within a range of +/− 5 years. A diagnosis of BD was confirmed using the Diagnostic and Statistical Manual of Mental Disorders (DSM-IVTR) [45] 40 and the Structured Clinical Interview by an experienced psychiatrist; euthymia was defined using the Hamilton Depression (HDRS-21 < 8) and Young Mania (YMRS < 7) Rating Scales at both medical screening and MRI scanning. Additionally, anxiety symptoms were assessed using the Hamilton Anxiety rating scale (HARS < 18). Exclusion criteria included neurological disorders, learning disability, comorbid misuse of substance/alcohol and of axis-1 disorders, history of head injury resulting in loss of consciousness for >5 min or a history of oral steroid use in the previous 3 months, unstable respiratory conditions, or history of cardiovascular events. Healthy controls had no personal history of a psychiatric illness or history of first-degree relatives (DSM-IV, Non-patient edition). A licensed pharmacist reviewed drug interactions with the cholinomimetic agent used in the study prior to administration. Ethical approval for the study was granted by the University College Hospital Galway, St. James’s Hospital Dublin and Mullingar CRECS, and participants gave written fully informed consent.

### Mood and word salience rating scale

The Profile of Mood States (POMS) questionnaire, the Visual Analogue scale [46] (VAS) and a word salience rating scale were administered before and after MRI scanning. Data on both were missing in one BD and one control participant. The POMS consisted of 65 emotionally valent words rated on a scale of 1, ‘not at all’ to 5, ‘extremely’. POMS scores formed four independent factors (Supplementary Table 1; Supplementary Fig. 1) using a principal component analysis, namely ‘depressed’ (*r* range:0.88–0.52), ‘fatigued’ (*r*:0.82–0.52), ‘positive’ (*r*:0.75–0.50) and ‘irritable’ (*r*:0.87–0.54). The VAS scores were found to encompass in the POMS scores entirely in terms of variance. Additionally, ﻿subjects subjectively rated the salience of previously validated [47] 79 emotionally valenced words on a scale of ‘very positive’ to ‘very negative’. Repeated measure Multivariate Analysis of Co-Variance (MANCOVA) with fixed factors diagnosis and challenge was carried out for group comparisons (*p* < 0.05). All statistical analyses were carried out using the Statistics Package for Social Sciences (SPSS v23, IBM, New York, USA).

### Emotion recognition and inhibition fMRI task

An emotion-inhibition fMRI task was chosen to assess regional functional activation involved in valence assessment of emotional content [48, 49], consisting of 180 pseudo-randomized trials belonging to two groups, emotion-recognition and emotion-inhibition trials, and three emotional valences for a total of six event types (International Affective Picture System database, IAPS [50]). Two unique batches of images matched for the degree of salience of each valence were used before and after physostigmine or placebo administration, resulting in sixty unrepeated pictures in each valence category (mean time of fMRI task events = 1.5 s). For further details on the fMRI paradigm and behavioural data acquisition refer to Supplementary Material.

We examined baseline behavioural performance (accuracy and reaction time) in BD relative to controls on placebo (MANCOVA, fixed factor diagnosis, covarying for age and gender, *p* < 0.05); and after physostigmine administration. Comparisons included the interaction between drug treatment, physostigmine or placebo, and diagnostic groups; and the effect of drug treatment within each diagnostic group (repeated measure MANCOVA, fixed factors diagnosis and challenge, covarying for age and gender; *p* < 0.05). *Post-hoc* investigations were carried out on the significant group differences (one-way ANOVA; *p*_FDR-corrected_ < 0.05;).

### Image acquisition & processing

MRI scanning was performed at the Welcome Trust Health Research Board National Centre for Advanced Medical Imaging (CAMI) at St. James’s Hospital Dublin, Ireland, using a 3 Tesla Achieva scanner (Philips, The Netherlands). High-resolution 3D T1-weighted turbo field echo magnetization-prepared rapid gradient-echo (MPRAGE) sequence using an eight-channel head coil (TR/TE = 8.5/3.046 ms, 1 mm^3^ isotropic voxel size, slice thickness = 1 mm, 160 slices) and a spin-echo EPI sequence (TR/TE = 2000/35 ms, 3 mm^3^ voxel size, slice thickness = 4.8, 37/38 slices) were acquired. Images were inspected for artefacts and motion; specifically, functional images were visually inspected and corrected for signal dropout and excessive head movement (translation and rotation; range <3 mm or +/−3°).

Functional MRI data were analyzed using SPM12 (http://www.fil.ion.ucl.ac.uk/spm). Images underwent slice-timing to TR/2, realignment to the first volume for motion correction, co-registration of the structural image to the mean of the motion corrected images using a 12-parameter affine transformation. Spatial normalization to 1 mm^3^ and smoothing (8 mm FWHM Gaussian kernel) were performed.

### Statistical analyses

First level *t*-test analyses using six contrasts, were defined for each fMRI scanning session including one contrast to assess the overall effect of the scanning session, two contrasts to investigate main effects of trial type (emotion recognition and emotion inhibition), and three to account for the different emotional valences (neutral, negative and positive). Movement parameters were used as regressors in the first level analysis; times at which questions were answered (onsets) were also accounted for. Second level T-contrasts included baseline comparison between diagnostic groups (i.e., BD-placebo *vs*. HC-placebo); further comparisons included the interaction between drug treatment, physostigmine or placebo, and diagnostic groups (i.e., BD-physostigmine *vs*. HC-physostigmine; BD-placebo *vs*. HC-placebo); and the effect of drug treatment within each diagnostic group (i.e., BD-physostigmine *vs*. BD-placebo; HC-physostigmine *vs*. HC-placebo). The investigation of emotion-inhibition trials presenting images of negative valence were prioritized in this study to test our a priori hypothesis that subjects with BD would display reduced capacity for inhibitory regulation of muscarinic-cholinergic neurotransmission upon stimulation of the cholinergic system with physostigmine relative to placebo and control groups. Age and gender were included as covariates in all the second level analyses. The threshold for significance was set as *p*_FWE-corr_ < 0.05 in clusters greater than 10-voxels in extent. The Brodmann Atlas [51] was used to localize the significant areas in Talairach space. Exploratory analyses were carried out *post-hoc* to investigate the contribution of SNP rs324650 on the main cluster findings. Thus, study participants underwent DNA genotyping from saliva samples and were grouped based on their allele frequency for SNP rs324650 (refer to Supplementary Material).

## Results

### Participants clinical and demographic characteristics

The imaging analysis featured a total of 33 participants with BD type I (*n* = 29) and type II (*n* = 4) and 50 controls aged 18–64; groups were matched for age and gender and education level (Table 1). Mood scores were available and examined for 46 participants with BD type I (*n* = 41) and type II (*n* = 5) aged 20–64, and 55 age and gender-matched controls. A total of 18 participants (*N* = 13 BD, *N* = 5 controls) were not included in the imaging analysis relative to the mood analysis. This was due to technical port errors in recording the fMRI behavioural data and a portion of study participants being removed from the scanner prior to completion of the first and/or second fMRI task due to nausea, one of the known peripheral side effects of physostigmine. For those who did complete imaging, the majority of BD participants were euthymic at the time of scanning (64%); *n* = 12 subjects displayed mild-to-moderate signs and symptoms (Table 1), and we indicate below that removing these subjects failed to alter the findings. Neither mood, as rated using the POMS-derived four factors depressed, fatigued, positive and irritable (F(4,90) = 0.332, *p* = 0.856), nor word salience (F(3,75) = 1.546, *p* = 0.210) differed significantly at baseline in the BD group relative to controls. Following physostigmine, these mood factors and word salience were not significantly altered relative to the placebo group and showed no interaction with diagnosis (POMS: F(4,90) = 0.850, *p* = 0.497; word salience: F(3,75) = 1.828, *p* = 0.149). Genotype-based recruitment within this sample was challenging in particular for the minor allele (TT genotype; Supplementary Table 2) and is considered hereafter as a *post-hoc* factor to inform future analyses.

**Table 1:** Clinical and socio-demographics.

	Healthy control	Bipolar disorder	Statistical comparison
Number of participants	50	33	
Gender, Male/Female (N)	23/27	18/15	χ^2^(1) = 0.581, *p* = 0.446
Age (Years)	40.1 ± 13.6	40.6 ± 11.5	*U* = 828.5, 0.974
Male/Female, mean ± SD	41.1 ± 13.5/39.3 ± 14.0	36.4 ± 10.7/45.4 ± 10.7	F(3,79) = 1.511, *p* = 0.218
Challenge, Placebo/Physostigmine (N)	9/41	8/25	χ^2^(1) = 0.476, *p* = 0.490
HDRS, mean ± SD (score)	1.0 ± 1.7	8.1 ± 7.6	*U* = 1430, *p* < 0.001*
median	0	5	
range	0–7	0–28	
HARS, mean ± SD (score)	0.7 ± 1.7	5.8 ± 6.8	*U* = 1319, *p* < 0.001*
median	0	3	
range	0–8	0–27	
YMRS, mean ± SD (score)	0.7 ± 1.5	2.2 ± 2.5	*U* = 1132.5, *p* = 0.001*
median	0	2	
range	0–6	0–10	
Age of onset (years)	–	24.7 ± 7.8	–
mean ± SD			
Illness duration (years)	–	15.0 ± 9.2	–
mean ± SD			
Level of education			
Median (score)	6	5	χ^2^(5) = 10.044, *p* = 0.074
Range	2–7	2–7	
Handedness, right/left-handed (N)	46/4	29/4	χ^2^(51) = 52.6, *p* = 0.413
Medication class (Frequency, N)	–	2	–
No medication			
Mood stabilizers	–		–
lithium (0.6–1.2 g/day)		3	
sodium valproate		9	
lamotrigine		7	
combination		9	
Antidepressants	–	9	–
Antipsychotics	–	25	–
Benzodiazepine	–	2	–
Other Psychotropics	–	6	–

Data is reported for the imaging sample. χ^2^ = Chi-squared test; *t* = independent sample *T*-test; *U* = Mann–Whitney U. *n* = 12 subjects with BD had a HDRS > 8; *n* = 4 subjects had a diagnosis of BD type II; age of onset is available for *n* = 29 subjects with BD. A total of 18 participants (*N* = 13 BD, *N* = 5 controls) were not included in the imaging analysis relative to the mood analysis. This was due to technical port errors in recording the fMRI behavioural data and a portion of study participants being removed from the scanner prior to completion of the first and/or second fMRI task due to nausea, one of the known peripheral side effects of physostigmine.

### Brain functional activation & emotion recognition and inhibition

The cholinergic challenge using physostigmine (1 mg) leads to increased functional activation in the posterior cingulate cortices during the inhibition and recognition of negative emotions and affects behavioural performance in BD (without altering mood) relative to healthy controls. While patients’ behavioural performance following physostigmine was overall lower than that of controls, physostigmine normalized processing ability (improved accuracy and reaction time) in the BD group during the inhibition of negative emotions, relative to placebo; this effect was associated with cingulate cortex overactivation in BD. We describe these results in detail below, starting with a baseline assessment of behavioural and imaging data in patients relative to controls, followed by an interaction analysis between drug allocation (physostigmine or placebo) and diagnostic group, and, lastly, physostigmine effects within each diagnostic group. The investigation of emotion-inhibition trials presenting images of negative valence were prioritized in this study to test our a priori hypothesis.

### Baseline behavioural and imaging analysis

At baseline, prior to physostigmine administration, we detected impaired emotion processing in BD relative to the control group overall (F(36,210) = 1.662, *p* = 0.015). In particular, having BD was associated with impaired accuracy (*p* = 0.012) and longer time to response (*p* = 0.029) in recognition but not inhibition (*p* = 0.164, *p* = 0.544) of negative emotions. Recognition or inhibition in the context of positive emotion did not differ significantly between diagnostic groups. Impaired recognition of negative emotion was associated with lower right caudate activation (*T* = 6.21, *p* < 0.001; Supplementary Fig. 2) and despite being as accurate as controls on the task when inhibiting a negative emotion, this was associated with lower activation of the left subgenual cingulate cortex (BA24, *T* = 5.39, *p* < 0.001; Supplementary Fig. 2).

#### Interaction between drug treatment and diagnostic group on behavioral and imaging data

**Fig. 1:**
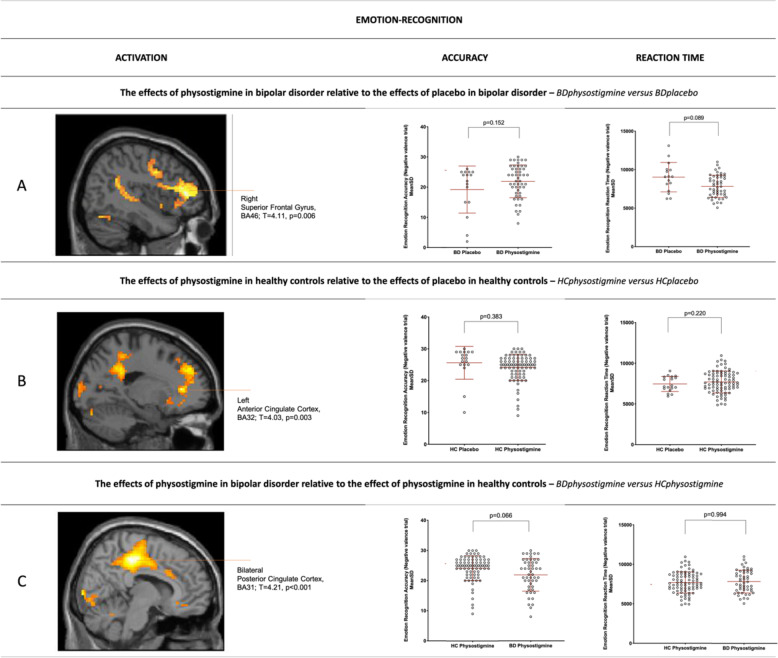
Physostigmine effects in Bipolar Disorder and Healthy Controls during the recognition of negative emotions. Panel shows the effects of physostigmine on functional activation and behavioural performance during the recognition of negative emotions. **A** increased activation in the right superior frontal gyrus (BA46, *T* = 4.11 *p* = 0.006) in BD receiving physostigmine relative to placebo; **B** decreased activation of the left anterior cingulate cortex (BA32, *T* = 4.03 *p* = 0.003) in controls receiving physostigmine relative to placebo. **C** increased activation within the bilateral dorsal cingulate cortices in BD receiving physostigmine relative to controls (BA31, *T* = 4.11, *p* < 0.001). Behavioural performance (accuracy and reaction times) remained unchanged (**A**–**C**). *BD* Bipolar Disorder; *HC* Healthy Controls. *Phys* Physostigmine; *p_FWE-corr_ < 0.05; 10 voxel-cluster.

**Fig. 2:**
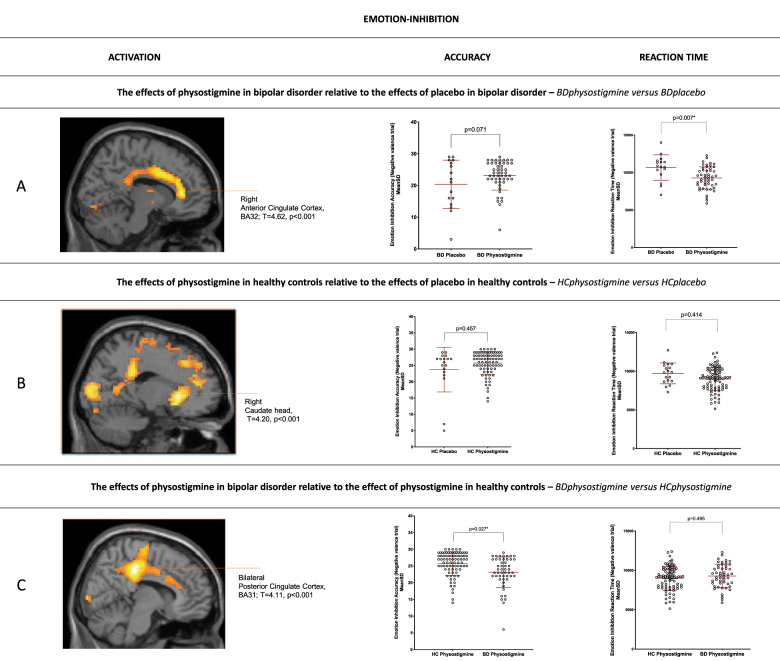
Physostigmine effects in Bipolar Disorder and Healthy Controls during the inhibition of negative emotions. Panel shows the effects of physostigmine on functional activation and behavioural performance during the inhibition of negative emotions. **A** increased activation in the right anterior cingulate cortex (BA32, *T* = 4.62 *p* < 0.001) in BD receiving physostigmine relative to placebo; and improved behavioral performance in terms of reaction times (*p* = 0.007); **B** decreased activation of the right caudate (*T* = 4.20 *p* < 0.001) in controls receiving physostigmine relative to placebo. **C** increased activation within the bilateral dorsal cingulate cortices (BA31, *T* = 4.11, *p* < 0.001) and improved accuracy in BD receiving physostigmine relative to placebo (*p* = 0.027), although still statistically lower than that of controls. *BD* Bipolar Disorder; *HC* Healthy Controls. *Phys* Physostigmine; *p_FWE-corr_ < 0.05; 10 voxel-cluster.

Physostigmine impacts emotion processing and cingulate activation differently in bipolar disorder relative to healthy controls: We investigated whether physostigmine/placebo administration would impact brain functional activation and/or behavioral performance in BD relative to controls. Overall, in accuracy and response times on recognition and inhibition (Supplementary Table 3) we detected an effect of diagnosis (F(12,66) = 3.097, *p* = 0.002), no effect of physostigmine (F(12,66) = 1.178, *p* = 0.317), and a significant diagnosis-by-physostigmine interaction (F(12,66) = 1.931, *p* = 0.046; Supplementary Table 3). This interaction showed that after physostigmine, BD behavioral performance (accuracy) remained significantly lower than controls during the inhibition, but not recognition (*p* = 0.495), of negative emotions (*p* = 0.027; Fig. 2C). In contrast, prior to receiving the challenge, at baseline, accuracy did not differ between these groups during the inhibition (*p* = 0.224) or recognition (*p* = 0.994) of negative emotions. Furthermore, in BD-physostigmine administration increased activation in the bilateral dorsal cingulate cortices (BA31) and supramarginal gyri (BA40) during both recognition (*T* = 4.21, *p* < 0.001, Fig. 1C) and inhibition (*T* = 4.11, *p* < 0.001; Fig. 2C) of negative and positive (*T* = 3.45, *p* = 0.001, *T* = 4.60, *p* < 0.001) emotion compared to controls. The main behavioral and imaging findings were confirmed when excluding 9 BD subjects who did not meet criteria for euthymia (refer to Supplementary Material). *Post-hoc* exploratory analyses including allele frequencies for SNP rs324650 in BD and controls confirmed a statistical effect of physostigmine in the posterior cingulate cortex for the BD-TT group (*n* = 4) on inhibition of negative emotions, with increased activation in the left posterior cingulate cortex (BA23) relative to controls-AA (*n* = 11, *post-hoc*, *p* < 0.001), and bilaterally relative to BD-AT (*n* = 11, *post-hoc*, BA23; *p* = 0.0348).

#### Within-subject comparisons on behavioural and imaging data

Physostigmine effects in healthy controls: Among controls, physostigmine administration relative to placebo did not significantly alter the accuracy or response time in the recognition (Fig. 1B) or inhibition (Fig. 2B) of negative emotions. Despite this, recognition (*T* = 4.58, *p* < 0.001; Fig. 1B) was associated with reduced activation in the left anterior cingulate cortex (BA32), left supramarginal (BA40), right middle occipital gyri (BA19) and right cerebellum, and inhibition (*T* = 4.20, *p* < 0.001; Fig. 2B) with reduced activation of the right caudate and cerebellum; while following physostigmine both recognition and inhibition of negative emotion was associated with reduced activation of the left superior frontal gyrus (BA8).

Physostigmine effects in bipolar disorder: In contrast, among the BD group, physostigmine relative to placebo improved the accuracy and response time on inhibition (*p* = 0.071, *p* = 0.007, Fig. 2A) but not recognition (*p* = 0.152, *p* = 0.089, Fig. 1A) of negative emotion and this was associated with increased activation in the right anterior cingulate (BA32) and middle frontal gyrus (BA10) for inhibition (*T* = 4.62, *p* < 0.001; Fig. 2A) and right subgenual cingulate gyrus (BA24) and superior frontal gyrus (BA46) for recognition (*T* = 4.11, *p* = 0.006; Fig. 1A).

## Discussion

This study demonstrates the involvement of the regulation of the muscarinic-cholinergic neurotransmission in clinically relevant behavioural features of BD, emotion inhibition, relative to a control group and to placebo. At baseline, BD emotion processing was impaired, and their anterior cingulate cortex was under activated, relative to controls. Administration of cholinergic challenge physostigmine affected emotion processing differently in controls than patients, with overactivation observed within the anterior cingulate cortex in BD, and under-activation within the anterior cingulate cortex in controls. Relative to controls, intravenous administration of physostigmine 1 mg at steady state normalized behavioural performance in BD without significantly altering mood (defined by factors depressed, fatigued, irritable, or positive), and was associated with cingulate cortex overactivation in patients, which significantly exceeded that seen in controls (Table 2).

**Table 2:** Summary of main study findings.

	Behavioral findings	Imaging findings
Baseline		
Emotion inhibition	↓ Accuracy; ↑ Reaction time	↓rh Caudate
Emotion recognition	–	↓lh Subgenual Cingulate Gyrus (BA24)
Physostigmine effects in BD (vs. Controls)		
Emotion inhibition	↓ Accuracy	↑ lh/rh Dorsal Cingulate Cortices (BA31); lh/rh Supramarginal Gyri (BA40)
Emotion recognition	–	↑ lh/rh Dorsal Cingulate Gyri (BA31); lh/rh Supramarginal Gyri (BA40)
Physostigmine effects in HC (vs. placebo)		
Emotion inhibition	–	↓ lh Superior Frontal Gyrus (BA8)
Emotion recognition	–	↓ lh Anterior Cingulate Cortex (BA32); lh Superior Frontal Gyrus (BA8); lh Supramarginal Gyrus (BA40); rh Middle Occipital Gyrus (BA19); rh Cerebellum
Physostigmine effects in BD (vs. placebo)		
Emotion inhibition	↑ Accuracy; ↑ Reaction time	↑rh Anterior Cingulate Cortex (BA32); rh Middle Frontal Gyrus (BA10)
Emotion recognition	–	↑rh Subgenual Cingulate Gyrus (BA24); rh Superior Frontal Gyrus (BA46)

Significant findings from the study are presented for functional activation and behavioural performance for emotion inhibition and recognition trials of negative emotions. *BD* Bipolar Disorder; *HC* Healthy Controls; “-” = not significant.

Cannon and collegues [6] previously reported an inverse relationship between the saliency of affective words and whole-brain M_2_ distribution volume in BD, in line with the known role of the muscarinic-cholinergic system attributing salience to experiential stimuli [52]. We failed to record group differences in word salience upon muscarinic-cholinergic challenge in BD, possibly due to the lack of effect of physostigmine on mood at the given dosage, which we expected in this sample considering worsening of mood had been observed only at higher dosages in euthymic and actively depressed BD subjects [15, 16].

We reported differential behavioural response in BD relative to controls during the inhibitory regulation or shifting of attention away from emotional stimuli. We also showed that reduced capacity for inhibition of cholinergic neurotransmission in BD was mediated by functional changes within regions involved in ventromedial and ventrolateral routes to emotional and attentional control [1]. The *ventromedial route* includes the subgenual anterior cingulate cortex, which in task-based fMRI studies of BD has been shown to relate to internal emotion processing such as autobiographical mood induction [1]; and specifically in this study, this may relate to internal processes of emotion inhibition. The *ventrolateral route* includes the anterior cingulate cortex, which relates to tasks examining external emotion control such as face processing [1], or in this study, image recognition. Anatomically, our imaging findings involve a constellation of functionally specialized subsystems of the brain responsible for the identification of emotionally salient stimuli, the generation of appropriate affective state, and the voluntary top-down regulation of emotional responses. Besides the prominent role of the cingulate gyri, other brain areas were recruited. For example, in the BD group at baseline before physostigmine administration, besides the cingulate, the caudate was also under activated when processing emotions relative to controls (Supplementary Fig. 2). The caudate nucleus is one of the areas where emotional states and behaviours originate [32]; when considered with its under-activation during emotion recognition in BD, it suggests patients may not be able to successfully focus on the emotional aspects of a stimulus and that stronger top-down regulation of regions originating emotional response may be needed for attention allocation to emotional stimuli, relative to controls. Following physostigmine in the BD group, in addition to the cingulate cortices (Fig. 2), the superior and middle frontal gyri were over-activated (Fig. 1); these regions collectively participate in ventromedial routes to emotional control and may relate to the deficient regulation of the emotional response elicited by the picture presented, and thus relate to biased valence processing seen in BD. In controls, following physostigmine, besides the cingulate cortices (Fig. 2), parieto-occipital regions were under activated during emotion inhibition, presenting an extensive inhibitory neurocircuitry functional model associated with emotion processing in healthy controls.

In summary, in controls, physostigmine induced reductions in cingulate functional activity, while emotion processing remained broadly unchanged, may reflect an intact autoreceptor system response to cholinergic challenge in this group. Contrastingly in BD, physostigmine induced increased activation within the cingulate cortex suggests a molecular deficit in this group, in line with the proposed reductions in inhibitory capacity seen in BD [29]. Of note, Cannon and colleagues [29] reported that it was not only in the cingulate cortex that the M_2_ receptors were reduced in BD; however, the ACC was the region displaying the statistically strongest differences [29]. While the cingulate cortex role within networks involved in emotion processing deficits is well established in BD both anatomically and functionally [1, 53], the neurotransmitter systems involved in these impairments, and more generally the contribution of these systems to mood dysregulation and cognitive control in psychiatry, are less investigated.

A preliminary look into the role of the rs324650 SNP in muscarinic-cholinergic function in BD following physostigmine administration showed greater activation in the posterior cingulate cortices during the inhibition of negative emotions in the BD-TT genotype group, relative to BD-AT and controls-AA; in line with reduced capacity for inhibition of cholinergic neurotransmission seen overall in BD in this study. Cannon and colleagues [30] previously showed that the TT polymorphism in BD was linked with a higher risk of attempting suicide, poorer social–occupational function and greater cognitive impairment. Future studies including further clinical and behavioural variables should investigate whether these deficits may reflect poorer cognitive reserve for BD subjects who are homozygous for the T allele. The effect of genetic variance in rs324650 on M_2_-receptor binding in the cingulate cortices in BD has been previously described [30], with BD-TT genotype showing the most significant reductions in M_2_-receptor binding (lower M_2_-receptor distribution volume) relative to BD-AT and controls-TT. Considering this receptor plays a major role in the regulation of ACh release, a genetic variation within CHRM2 that alters the function of this autoreceptor could, in turn, alter the capacity for regulation of cholinergic neurotransmission and thus exert widespread effects on a variety of emotional and cognitive domains and their interaction, particularly in BD.

It is to be noted that our BD sample was taking a range of medications at the time of scanning. ﻿Their effect and interaction on physostigmine were beyond the scope of this study. Although we cannot exclude medication effects on our findings, a recent review of medication effects in neuroimaging studies in BD suggests that medication impact is higher for structural (volumetric) MRI than it is for diffusion or functional MRI [54].

In closing, physostigmine induced increase in cingulate cortex functional activation during emotion processing in BD may be considered a marker of cholinergic sensitivity and emotion dysregulation in this illness. When considered together with the normalization of emotion processing ability seen in the BD group in this study, these physostigmine induced effects may represent a valid dose-sensitive target for core symptom management in BD. Recently, the relative increase in acetylcholine concentrations and cholinergic signalling dysregulation in BD has been a potential target for muscarinic antagonists such as scopolamine [55], which has been proven a fast-onset and sustained antidepressant in medication naïve and treatment-resistant BD [56]; however, its dose-response profile and time-course antidepressant effects and impact on cognition will require further investigation. We also report preliminary evidence of cingulate involvement in a subgroup of BD participants with the TT genotype of SNP rs324650. While preliminary evidence suggests we may be coming closer to being able to exploit the cholinergic hypothesis of mood [16, 55] to ameliorate core features of BD illness, further clinical trials using muscarinic-cholinergic antagonists are warranted and future research should strive to incorporate a genomics approach to define muscarinic-cholinergic sensitivity to potentially detect those individuals more likely to benefit from these agents.

## Supplementary information

Supplemental Material
