# Supplementary material for: Normalization of impaired emotion inhibition in bipolar disorder mediated by cholinergic neurotransmission in the cingulate cortex
Source: Neuropsychopharmacology. 2022 Jan 19;47(9):1643–51. doi: 10.1038/s41386-022-01268-7 (PMC9283431; doi:10.1038/s41386-022-01268-7)
Supplement: Supplementary file 1 — Supplemental Material [file 41386_2022_1268_MOESM1_ESM.docx]

**Supplementary Information**

*Genotyping*

Each study participant gave written fully informed consent to provide their saliva samples. Saliva samples were collected using Oragene DNA OG-500 kit^1^ and underwent DNA purification using 120 µL PT-L2P^2^ solution, 100% and 70% ethanol, and ﻿100 µL of 1% TrisEDTA for rehydration. DNA quantification was carried out using ﻿BR-DNA assay and ﻿Qubit 2.0 fluorimeter^3^. Genotyping was performed using TaqMan (fluorogenic 5’ nuclease)^4^ assay for SNP rs324650 and ABI PRISM 7900 sequence detection system^5^. Allele discrimination was subjected to polymerase chain reaction (PCR) and results were visualized using StepOneplus 2.3 software^5^. ﻿Genotypes were typed on reverse strand (3’ – 5’) of DNA as per previous publication^6^. Genotype-based recruitment within this sample was challenging in particular for the minor allele (TT-genotype) and is considered as a *post-hoc* factor to inform future analyses. For example, of the BD participants with the TT-genotype, only 4 received physostigmine and 2 received placebo (genotype groups were matched for age and gender, **Supplementary Table 2**).

*Supplemental analyses removing non-euthymic subjects*

Supplemental analyses excluding nine non-euthymic BD subjects showed moderate depressive signs and symptoms, which confirmed our main findings Overall, there was a statistically significant challenge-by-diagnosis interaction on behavioral measures following physostigmine (F(12,57)=2.137, p=0.028) and specifically on the inhibition of negative emotions (F(1,68)=5.438, p=0.023) with a trend for reduced accuracy seen in BD on physostigmine (n=16) relative to controls on physostigmine (n=41) (*post-hoc*, p=0.055). Removal of non-euthymic subjects confirmed over-activation in the right posterior cingulate cortex during inhibition (BA23; *post-hoc,* T=4.13, p<0.001) and recognition (BA31; *post-hoc,* T=4.48, p<0.001) of negative emotions, and inhibition of positive emotions (BA31; *post-hoc,* T=4.03, p<0.001).

1. DNA Genotek. OG-500 DNA from Saliva For In Vitro Diagnostic Use For collection of human DNA Collect superior samples for your genetic analysis and testing. *Cancer Epidemiol Biomarkers Prev* **19**, 794–8 (2010).

2. DNA Genotek. PT-L2P Protocol to maximize DNA recovery. 6354 (2014).

3. Life technologies. Qubit® 2.0 Fluorometer. *Invitrogen* **9**, 2417–2427 (2010).

4. Applied Biosystems. TaqMan SNP Genotyping Assays USER GUIDE (Publication Number MAN0009593). 72 (2017).

5. Applied Biosystems. Applied Biosystems StepOne^TM^ and StepOnePlus^TM^. *Appl. Biosyst. StepOne^TM^ StepOnePlus^TM^ Real-Time PCR Syst.* 1–292 (2010).

6. Cannon, D. M. *et al.* Genetic variation in cholinergic muscarinic-2 receptor gene modulates M2 receptor binding in vivo and accounts for reduced binding in bipolar disorder. *Mol. Psychiatry* **16**, 407 (2010).

**Tables and Figures**

**Supplementary Table 1. Principal Mood Components of the POMS Rating Scales.**

**Supplementary Table 1 Legend:** The table illustrates the first 4 components extracted from the principal component analysis comprising each factor alongside their correlation (r) values. Only correlations greater than 0.5 are considered and reported.

**Supplementary Table 2. Supplemental demographics across genotypes.**

**Supplementary Table 2 Legend:** *One BD participants was not genotyped, resulting in total sample size N=82. Genotypes were typed on reverse strand (3’ – 5’) of DNA.

**Supplementary Table 3. Effect of time, diagnosis and physostigmine on behavioral performance accuracy and reaction time in bipolar disorder relative to healthy controls.**

**Supplementary Table 3 Legend****:** With regards to behavioral performance, upon physostigmine, repeated measures MANCOVA showed a main effect of scan time (F(12,66)=2.361, p=0.014), a time-by-diagnosis interaction (F(12,66)=3.097, p=0.002), no main time-by-challenge interaction (F(12,66)=1.178, p=0.317), but a significant time-by-challenge-by-diagnosis interaction (F(12,66)=1.931, p=0.046). Accuracy (Accu) and reaction time (RT, s) are reported as Mean±SD and presented for both groups pre and post-infusion with cholinergic challenge physostigmine (1 mg) across all trial types and emotional valences. HC = Healthy Controls; BD = Bipolar Disorder; Accu = Accuracy; RT = reaction time; Emo-rec= Emotion-recognition trial; Emo-inhib= Emotion-inhibition trial. Repeated measures MANCOVA fixed factor diagnosis and challenge, covarying for age and gender, **p*<0.05.

**Supplementary Figure 1. Principal Component analysis scree plot.**

**Supplementary Figure 1 Legend:** The first point of inflection was observed at component number 4 explaining for the majority (51.9%) of the variance in the data. A second point of inflection was observed at component number 7, however this was responsible for explaining little variance (12.5%) compared to component 4. Therefore, only data at the point of inflection at component 4 were considered.

**Supplementary Figure 2. Baseline effects in Bipolar Disorder relative to healthy controls in emotion-recognition and emotion-inhibition.**

**Supplementary Figure 2 Legend:** Top: reduced activation in the right caudate was recorded in bipolar disorder relative to controls (emotion-recognition: T=6.21, p<0.0001) and impaired behavioral performance (accuracy: p=0.012; reaction time: p=0.029). Bottom: Prior to cholinergic challenge, reduced activation in the left subgenual cingulate cortex (BA24; T=5.39, p<0.001) was observed in bipolar disorder relative to controls on placebo and impaired behavioral performance, although not significant (accuracy: p=0.164, reaction time: p=0.544).

Supplementary Table 1. Principal Mood Components of the POMS Rating Scales.

| PCA components | | | | | | | |
| --- | --- | --- | --- | --- | --- | --- | --- |
| 1 |  | **2** |  | **3** |  | **4** |  |
| DEPRESSED | **Correlation** | **FATIGUE** | **Correlation** | **POSITIVE** |  | **IRRITABLE** | **Correlation** |
| Miserable | 0.877 | Exhausted | 0.815 | Considerate | 0.75 | Angry | 0.873 |
| Desperate | 0.803 | Weary | 0.701 | Cheerful | 0.729 | Bad Tempered | 0.854 |
| Gloomy | 0.801 | Bushed | 0.644 | Good natured | 0.728 | Furious | 0.81 |
| Hopeless | 0.789 | Sluggish | 0.63 | Helpful | 0.726 | Grouchy | 0.734 |
| Lonely | 0.737 | Fatigued | 0.625 | Full of Pep | 0.682 | Annoyed | 0.712 |
| Bitter | 0.693 | Unable to Concentrate | 0.589 | Sympathetic | 0.635 | Peeved | 0.689 |
| Worthless | 0.624 | Helpless | 0.587 | Alert | 0.606 | Listless | 0.667 |
| Blue | 0.624 | Muddled | 0.573 | Friendly | 0.599 | Resentful | 0.58 |
| Discouraged | 0.615 | Worn Out | 0.57 | Relaxed | 0.575 | Ready to Fight | 0.539 |
| Guilty | 0.614 | Confused | 0.542 | Trusting | 0.57 |  |  |
| Unhappy | 0.608 | Uneasy | 0.515 | Energetic | 0.554 |  |  |
| Panicky | 0.584 |  |  | Efficacious | 0.55 |  |  |
| Restless | 0.58 |  |  | Lively | 0.532 |  |  |
| Uncertain about things | 0.552 |  |  | Carefree | 0.525 |  |  |
| Sad | 0.544 |  |  | Active | 0.503 |  |  |
| Helpless | 0.537 |  |  |  |  |  |  |
| Resentful | 0.53 |  |  |  |  |  |  |
| Terrified | 0.518 |  |  |  |  |  |  |

**Supplementary Table 2. Supplemental demographics across genotypes.**

|  | ***Healthy Controls*** | | | ***Bipolar Disorder*** | | | ***Total, N**** | ***Chi-square (χ^2^),***  ***p-value*** |
| --- | --- | --- | --- | --- | --- | --- | --- | --- |
|  | ***TT*** | ***AT*** | ***AA*** | ***TT*** | ***AT*** | ***AA*** |  |  |
| ***Physostigmine/Placebo, N*** | 10/3 | 20/3 | 11/3 | 4/2 | 11/5 | 9/1 | 82 | 3.12, 0.79 |
| ***Male/female*** | 5/8 | 14/9 | 4/10 | 3/3 | 9/7 | 6/4 | 82 | 4.67, 0.59 |

**Supplementary Table 3. Effect of time, diagnosis and physostigmine on behavioral performance accuracy and reaction time in bipolar disorder relative to healthy controls.**

|  | *Pre cholinergic challenge* | | | | *Post cholinergic challenge* | | | | *Effect of Scan Time* | *Interaction between Scan Time and Diagnosis* | *Interaction between Scan Time, Diagnosis and Challenge* |
| --- | --- | --- | --- | --- | --- | --- | --- | --- | --- | --- | --- |
|  | ***HC*** | | ***BD*** | | ***HC*** | | ***BD*** | |  |  |  |
|  | ***Placebo*** | ***Physostigmine*** | ***Placebo*** | ***Physostigmine*** | ***Placebo*** | ***Physostigmine*** | ***Placebo*** | ***Physostigmine*** |  |  |  |
|  | *Mean±SD* | *Mean±SD* | *Mean±SD* | *Mean±SD* | *Mean±SD* | *Mean±SD* | *Mean±SD* | *Mean±SD* | *F, p* | *F, p* | *F, p* |
| *Accu Emo-rec Neutral* | 17.22±5.83 | 18.98±5.48 | 17.13±5.79 | 16.36±6.55 | 18.11±6.01 | 21.51±4.16 | 21.13±4.58 | 20.16±6.90 | 1.569, 0.214 | 3.731, 0.057 | 0.996, 0.321 |
| *Accu Emo-rec Negative* | 26.00±4.58 | 23.80±4.28 | 18.63±8.18 | 22.16±4.56 | 25.22±5.93 | 24.54±4.06 | 19.75±7.89 | 21.68±6.23 | 0.105, 0.747 | 0.11, 0.741 | 2.284, 0.135 |
| *Accu Emo-rec Positive* | 25.56±3.36 | 21.73±3.36 | 20.88±6.71 | 22.96±3.54 | 22.78±6.22 | 17.85±5.89 | 17.13±6.03 | 18.52±5.92 | 7.182, 0.009* | 0.347, 0.557 | 0.075, 0.785 |
| *Accu Emo-inhib Neutral* | 25.89±4.43 | 25.83±3.84 | 18.00±8.83 | 23.68±5.22 | 23.78±7.58 | 25.76±3.06 | 22.25±5.55 | 24.72±4.67 | 1.656, 0.202 | 16.062, 0.00014* | 9.875, 0.002* |
| *Accu Emo-inhib Negative* | 22.89±7.13 | 24.51±4.19 | 17.50±9.02 | 21.92±4.72 | 24.56±6.86 | 26.95±2.21 | 23.25±4.86 | 24.32±4.18 | 0.186, 0.668 | 4.505, 0.037* | 5.855, 0.018* |
| *Accu Emo-inhib Positive* | 21.89±5.97 | 24.37±4.53 | 17.13±8.36 | 21.64±5.69 | 22.22±5.93 | 24.00±2.00 | 20.50±6.78 | 22.24±3.63 | 1.432, 0.235 | 4.535, 0.036* | 1.916, 0.17 |
| *RT Emo-rec Neutral* | 1.00±0.13 | 0.92±0.17 | 1.04±0.12 | 0.94±0.17 | 0.93±0.15 | 0.83±0.17 | 0.96±0.15 | 0.85±0.19 | 4.242, 0.043* | 0.008, 0.929 | 0.001, 0.971 |
| *RT Emo-rec Negative* | 0.75±0.09 | 0.79±0.14 | 0.95±0.20 | 0.79±0.13 | 0.74±0.09 | 0.75±0.13 | 0.86±0.18 | 0.78±0.16 | 3.192, 0.078 | 0.522, 0.472 | 5.60, 0.020* |
| *RT Emo-rec Positive* | 0.79±0.08 | 0.81±0.13 | 0.94±0.15 | 0.79±0.12 | 0.75±0.09 | 0.90±0.16 | 0.91±0.16 | 0.78±0.12 | 0.089, 0.766 | 1.594, 0.211 | 0.266, 0.608 |
| *RT Emo-inhib Neutral* | 0.95±0.08 | 0.88±0.15 | 1.02±0.17 | 0.95±0.16 | 0.89±0.12 | 0.83±0.14 | 0.98±0.18 | 0.88±0.18 | 0.5, 0.481 | 0.015, 0.904 | 0.753, 0.388 |
| *RT Emo-inhib Negative* | 1.00±0.10 | 0.95±0.14 | 1.10±0.17 | 0.98±0.13 | 0.94±0.16 | 0.85±0.15 | 1.04±0.18 | 0.89±0.16 | 4.12, 0.046* | 0.323, 0.571 | 0.025, 0.876 |
| *RT Emo-inhib Positive* | 1.00±0.11 | 0.94±0.14 | 1.00±0.19 | 0.95±0.13 | 0.89±0.12 | 0.81±0.14 | 0.98±0.18 | 0.86±0.18 | 2.192, 0.143 | 4.898, 0.030* | 1.067, 0.305 |

**Supplementary Figure 1. Principal Component analysis scree plot.**

| ***Component*** | ***Eigenvalue*** | ***Percent Variance Explained*** |
| --- | --- | --- |
| 1 | 18.74 | 28.83 |
| 2 | 6.839 | 39.352 |
| 3 | 4.642 | 46.493 |
| 4 | 3.502 | 51.881 |


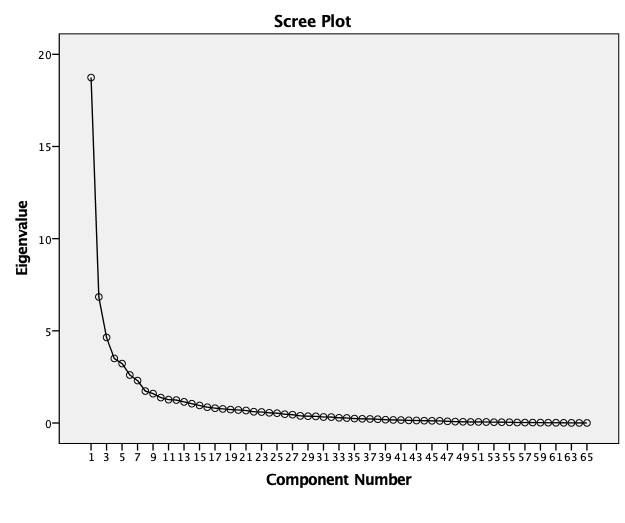


**Supplementary Figure 2. Baseline effects in Bipolar Disorder relative to healthy controls in emotion-recognition and emotion-inhibition.**
